# Bacterial Expression and Kinetic Analysis of Carboxylesterase 001D from *Helicoverpa armigera*

**DOI:** 10.3390/ijms17040493

**Published:** 2016-04-02

**Authors:** Yongqiang Li, Jianwei Liu, Mei Lu, Zhiqing Ma, Chongling Cai, Yonghong Wang, Xing Zhang

**Affiliations:** 1 Research and Development Centre of Biorational Pesticides, Northwest Agriculture and Forestry University, Yangling 712100, China; Yongqiangli@nwsuaf.edu.cn (Y.L.); lumei_1990@nwsuaf.edu.cn (M.L.); zhiqingma@nwsuaf.edu.cn (Z.M.); cchongling@nwsuaf.edu.cn (C.C.); zhxing1952@gmail.com (X.Z.); 2 Commonwealth Scientific and Industrial Research Organisation Land & Water Flagship, Canberra, ACT 2601, Australia; Jian-Wei.Liu@csiro.au

**Keywords:** *Helicoverpaarmigera*, carboxylesterase, pyrethroid, heterologous expression

## Abstract

Carboxylesterasesare an important class of detoxification enzymes involved in insecticide resistance in insects. A subgroup of *Helicoverpa armigera* esterases, known as Clade 001, was implicated in organophosphate and pyrethroid insecticide resistance due to their overabundance in resistant strains. In this work, a novel carboxylesterasegene *001D* of *H. armigera* from China was cloned, which has an open reading frame of 1665 nucleotides encoding 554 amino acid residues. We used a series of fusion proteins to successfully express carboxylesterase 001D in *Escherichia coli*. Three different fusion proteins were generated and tested. The enzyme kinetic assay towards 1-naphthyl acetate showed all three purified fusion proteins are active with a *K*cat between 0.35 and 2.29 s^−1^, and a *K*m between 7.61 and 19.72 μM. The HPLC assay showed all three purified fusion proteins had low but measurable hydrolase activity towards *β*-cypermethrin and fenvalerate insecticides (specific activities ranging from 0.13 to 0.67 μM·min^−1^·(μM^−1^·protein)). The enzyme was stable up to 40 °C and at pH 6.0–11.0. The results imply that carboxylesterase 001D is involved in detoxification, and this moderate insecticide hydrolysis may suggest that overexpression of the gene to enhance insecticide sequestration is necessary to allow carboxylesterases to confer resistance to these insecticides in *H. armigera.*

## 1. Introduction

The cotton bollworm, *Helicoverpa armigera*, is a major crop pest in many parts of the world [1,2,3,4]. It has rapidly developed resistance to organophosphate (OP) and pyrethroid insecticides [5,6,7,8]. Carboxylesterases (EC3.1.1.1) play a critical role in the detoxification of xenobiotic compounds and are implicated in pyrethroid and OP resistance in a wide variety of pest insect species [9,10,11,12,13,14]. Carboxylesterase-mediated metabolic resistance to OPs and pyrethroids can occur through two mechanisms. The first one involves mutations in the active site (oxyanion hole and acyl-binding pocket) of the enzyme that elevates the OP hydrolytic activity and reduces the activity towards carboxylesterase substrates, such as 1‑naphthyl acetate [15,16,17,18]. The second mechanism involves overexpression of unaltered carboxylesterases based on gene amplification or up-regulated transcription, allowing for effective sequestration of the insecticides instead of hydrolysis [19,20,21,22,23].

In *H. armigera*, a correlation between enhanced esterase activity and pyrethroid resistance was found in Australia, Asia, and Africa [4,23,24,25,26,27]. Understanding or combating esterase-mediated resistance is hindered by the fact that over 70 esterases may be present within lepidopteran genomes [28]. Thirty-nine putative paralogous carboxy/cholinesterase (CCE) sequences were identified from a susceptible *H. armigera* GR strain, and they were figured into a lot of subgroups according to a phylogenetic analysis [28]. In most cases, the overexpressed esterases in resistant strains remain unidentified at the molecular level, and their substrate spectrums and inhibitor profiles are unknown. In the most extensive study of *H. armigera* esterases derived from the susceptible GR strain, eight carboxylesterases belonging to the CCE Clade 001 were expressed in Sf9 insect cells, which showed tight binding to OPs and some hydrolytic activity towards OPs [29,30]. However, they all showed relatively low activities against nine cypermethrin and fenvalerate isomers [29,30]. In the resistant *H. armigera* YGF strain from China, two unique isozyme bands in native polyacrylamide gel electrophoresis (PAGE) were matched with four carboxylesterase genes, *001A*, *001D*, *001I*, *001J* by mass spectrometry analysis, shown to be overexpressed up to 90-fold, as compared to the susceptible SCD strain [23]. Additionally, proteomic and molecular analyses of the OP-resistant *H. armigera* strain (MonoR) from China also showed that the overexpression of six Clade 001 enzymes, including 001D, was associated well with monocrotophos resistance [12].They showed that carboxylesterases in *H. armigera* were involved in resistance to OP and pyrethroid insecticides via enhanced sequestration, favoured by overexpression. Nearly all published studies focusing on the hydrolytic activity of *H. armigera* carboxylesterases towards OP and pyrethroid insecticides used the enzymes heterologously expressed in insect Sf9 cells with the baculovirus expression system [29,30,31]. In other cases, the carboxylesterase E3 from *Lucilia cuprina* and the juvenile hormone esterase gene (*Nljhe*) from *Nilaparvata lugens* have also been expressed in the baculovirus expression system [15,32]. However, due to slow cell growth and low expression levels within eukaryotic cells, only limited amounts of the enzymes can be produced. As a consequence, all of the above studies have been restricted to catalytic studies of unpurified enzymes in raw cell extracts. In contrast, the *E. coli* system is a preferred expression system because of its extensive characterization and ease of handling [33]. Some insect esterases have been shown to be active when expressed in highly efficient bacterial systems, such as in *E. coli* [34,35,36]. In many other cases, however, problems with protein folding have failed to yield active enzymes after expression in bacterial cells. For example, 3 of the 14 *H. armigera* carboxylesterases from the susceptible GR strain showed no esterase activity when expressed in *E. coli*, using the Gateway-compatible expression vector with their native start and stop codons [37].

In this study, we sequenced a cDNA (*001D*) encoding a carboxylesterase from an insecticide-susceptible *H. armigera* strain from the Wuhan region of China (WH). In order to obtain active enzyme, we heterologously expressed three fusion proteins containing three different solubility/affinity tags in the *E. coli* cells. We purified each fusion protein and evaluated their hydrolytic activities towards a model substrate (1-naphthyl acetate, 1-NA) and two pyrethroid insecticides (*β*-cypermethrin and fenvalerate). We demonstrated that all three purified proteins of 001D were capable of metabolizing two real insecticide substrates. Moreover, the effect of temperature and pH on the enzyme activity was also explored, and the mechanism of 001D involved in pyrethroids resistance is further discussed.

## 2. Results

### 2.1. Cloning and Sequence Analysis

**Figure 1:**
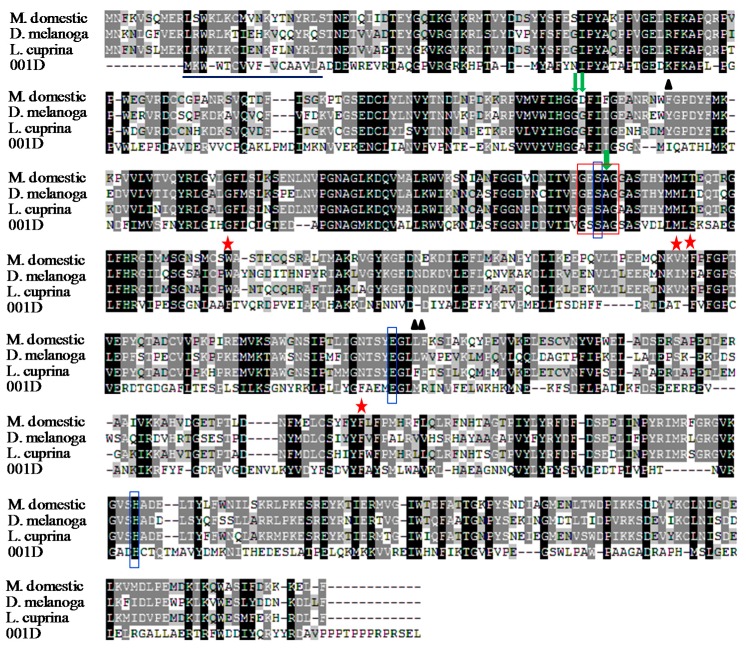
Alignment of the deduced amino acid sequences of 001D (GenBank^®^ accession number KT345935) from the *H. armigera* Wuhan (WH) strain with the previously reported insect carboxylesterase E7 (GenBank^®^ accession number AAD29685) from *Musca domestica*, аE7 (GenBank^®^ accession number NP_524261) from *Drosophila melanogaster* and the E3 (GenBank^®^ accession number AAB67728) from *L. cuprina*. The signal peptide sequences of 001D are underlined, the catalytic triad residues are vertically boxed in blue color, the highly conserved pentapeptide residue are boxed with red color, dark triangles indicate the anionic site, red asterisks indicate the acyl-binding pocket, green arrows mean the oxyanion hole.

**Figure 2:**
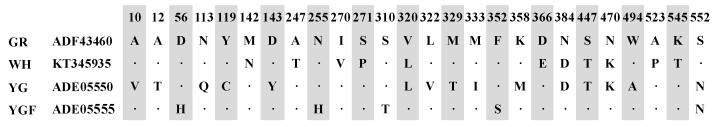
Comparison of amino acid differences of 001D from *H. armigera* between the WH, GR, YG, and YGF strains (GenBank^®^ accession numbers KT345935, ADF43460, ADE05550, and ADE05555). Amino acids the same as that of the GR strain are indicated with a dot. Numbering indicates the alignment number of different residue amino acids.

Thecarboxylesterase gene *001D* was cloned from a cDNA library of the midgut of *H. armigera* from the susceptible Wuhan (WH) strain. The novel cDNA sequence of *001D* (GenBank^®^ accession number KT345935) has an open reading frame of 1665 nucleotides, encoding 554 amino acid residues with a molecular weight of 62.6 kDa, and an isoelectric point of 5.27. It includes a signal peptide containing a cleavage site between the 16th and 17th amino acids. The alignment showed that carboxylesterase 001D has highly conserved residues of a catalytic triad (S202-H443-E330) and a pentapeptide termed the nucleophilic elbow (G200-S201-S202-A203-G204) (Figure 1). 001D also contains the other three subsites, including the leaving group pocket (M333-R334-I133), acyl pocket (F235-T287-F309), and oxyanion hole (G136-G137-A203) [18]. The characterization of the enzyme active site thus indicates that carboxylesterase 001D may have hydrolytic function. BLAST search with the amino acid sequence (Figure 2) showed that the carboxylesterase shared 98% similarity to the carboxylesterase 001D of the pyrethroid-susceptible GR strain (GenBank^®^ accession number ADF43460) from Australia [28]. Additionally, the amino acid sequence showed 96% and 97% similarity to the carboxylesterase of the pyrethroid-susceptible YG and resistant YGF strain (GenBank^®^ accession numbers ADE05550 and ADE05555) from China, respectively [23].

### 2.2. Purification of Recombinant Carboxylesterases and Western Blot Assay

**Figure 3:**
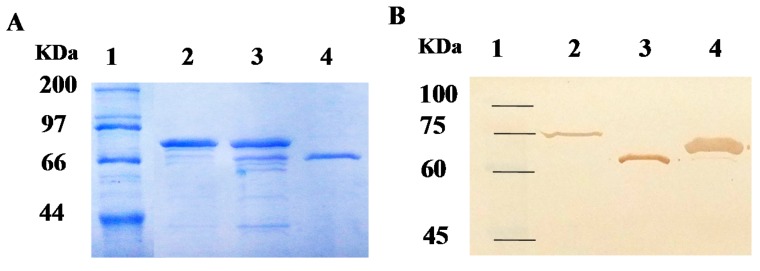
SDS-PAGE and Western blot analysis of expression and purification of recombinant 001D. (**A**) The samples were separated on 12% gels; and (**B**) Western blot analysis of recombinant carboxylesterase using anti-His tag antibody. Lane **1**: Protein ladder, Lane **2**: purified Trx/6×His/S-tag/001D from pET32a, Lane **3**: purified 6×His-tag/001D from pE1, Lane **4**: purified 6×His/S-tag/001D from pET30a.

The coding sequence of the *001D* gene without signal peptide was inserted in three types of expression vectors, pE1, pET30a, and pET32a, and expressed in the *E. coli* BL21 (DE3) strain. Three different fusion proteins, 6×His/001D (pE1), 6×His/S-tag/001D (pET30a), and Trx/6×His/S-tag/001D (pET32a) were purified using Ni^2+^-Nitrilotriacetic acid (NTA) resin (Figure 3). The purified recombinant enzyme 001D from pET32a and pE1 showed poor purity after affinity chromatography and gave more than two bands, but the purified 001D from pET30a showed only a single band of approximately 67 kDa on the SDS-PAGE, in good agreement with the theoretical molecular mass of 6×His/S-tag/001D (Figure 3A). The antibody recognized a single band from all three purified recombinant proteins as shown in Western blotresult (Figure 3B). By contrast with the 001D band from pET30a vector, a weak Western signal was detected for recombinant enzyme produced from pET32a and pE1 vectors, which should be the target bands, as it is identical with the theoretical molecular weight of the fusion proteins, Trx/6×His/S-tag/001D and 6×His-tag/001D (79 and 63 kDa), respectively. The concentration of the purified 001D from pET30a is 70 mg·L^−1^ based on Bradford assay, and it is pure as a single band. The concentration of both 001D from pET32a and pE1 are estimated from the Western blot by using GelQuantNET software from Biochem-Lab Solutions (University of California, San Francisco, CA, USA).

### 2.3. Enzymatic Activity of 001D towards an Artificial Carboxylester

Activities of the purified recombinant enzymes towards the 1-naphthyl acetate (1-NA) were determined and shown in Table 1. All three fusion proteins showed esterase activity towards 1-NA. Among the three fusion proteins, the recombinant enzyme of 001D from pET32a exhibited the highest catalytic efficiency towards 1-NA, with a *K*m of 7.61 μM and a *k*cat of 2.29 s^−1^, showing it has a high affinity and turn-over to the substrate compared to those of the recombinant 001D from pE1 and pET30a. The fusion protein of 001D from pE1 had a very poor affinity and low turn-over to the substrate. In contrast, the purified protein of 001D from pET30a showed moderate esterase activity towards 1-NA with a specific activity of 0.57 μM·s^−1^·(μM^−1^·protein). The results suggest that the three types of fusion tags have different effects on enzymatic activity of the carboxylesterase 001D, among them, the larger fusion tag, Trx-tag, has strongest enhancing effect on the esterase activity of 001D towards the model substrate.

### 2.4. Hydrolysis Activity of 001D towards Pyrethroid Insecticides

The specific activities of all the three purified recombinant enzymes against the pyrethroids were determined. As shown in Table 2, all three fusion proteins had low but measurable activities towards both the *β*-cypermethrin and fenvalerate substrates. The purified fusion proteins of 001D from pET32a showed relatively lower hydrolase activity towards the two real insecticides, as compared to the protein from pET30a. However, the specific activities of 001D from pET30a are higher, 0.41 and 0.67 μM·min^−1^·μM^−1^ towards the two pyrethroid substrates, respectively. For the *β*-cypermethrin substrate, the hydrolysis rate is 2-fold higher than that from pET32a and pE1. For the fenvalerate substrate, the hydrolysis rate is 2- and 5-fold higher than that from pET32a and pE1, respectively (Table 2). Although the specific activities varied when the 001D was tagged with different fusion tags, the results reveal that all of the three fusion proteins can metabolize the two real insecticides, thus indicating that the carboxylesterase 001D is involved in detoxification of pyrethroid insecticides in the susceptible WH strain.

### 2.5. Effects of Temperature and pH on the Enzyme Activity

**Figure 4:**
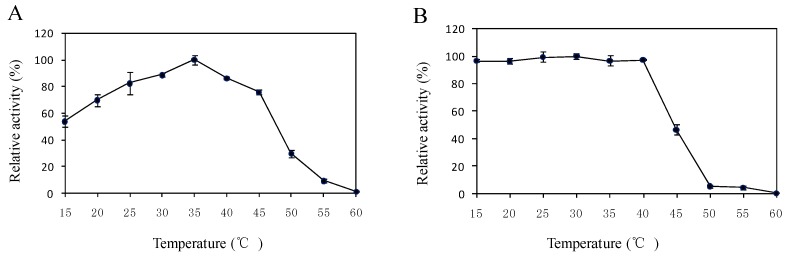

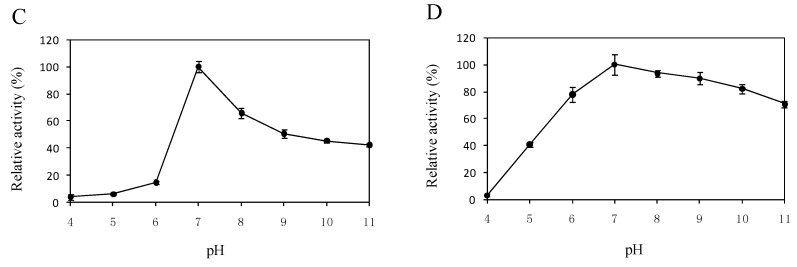
Effect of temperature and pH on enzyme activity of purified 001D from pET32a. (**A**) Temperature optimum; (**B**) Thermostability; (**C**) pH optimum; and (**D**) pH stability. In each case, the highest activity was set at 100%. Each treatment was done in triplicate.

As shown in Figure 4A, the optimal temperature for enzyme activity was 35 °C. The relative activities were more than 69.9% in the temperature range of 20 °C–45 °C. The thermo stability assay revealed that the enzyme was stable up to 40 °C, and 46.8% of residual activity remained at 45 °C (Figure 4B). The enzyme lost activity when it was incubated at 60 °C for 1 h. The pH also had marked effects on the enzymatic activity. The enzyme showed the best activity at pH 7.0, which declined to 50.6% when the pH increased to 9.0 (Figure 4C). However, 001D was stable within the pH range from 6.0 to 11.0, and the remaining enzyme activity was more than 70% after 1 h (Figure 4D).

## 3. Discussion

There is a great interest in carboxylesterases due to their critical role in the detoxification of xenobiotic compounds, including OP and pyrethroid insecticides [9,10,11,18]. To understand the catalyzation function of carboxylesterase, it is essential to yield the highly pure protein *in vitro* or *in vivo*. Although the *E. coli* system is a very convenient expression system to obtain purified recombinant protein *in vitro*, it is usually unsuitable to be used for the expression of the recombinant protein from most eukaryotes, such as insects, plants, and animals, because of highly divergent codon usage and protein misfolding [38]. A variety of techniques were used to overcome problems associated with poor protein solubility [38,39,40]. In our present work, a novel carboxylesterase gene 001D from the susceptible *H. armigera* WH strain was cloned. The deduced amino acid sequence comprised the highly conserved catalytic triad, indicating that 001D can function as an active esterase. However, other subsites around the catalytic triad are more variable in different insects [41,42]. We further demonstrated that carboxylesterase 001D can be heterologously expressed with different fusion proteins in the *E. coli* cells. All three purified fusion proteins were active, and displayed hydrolase activities against the model substrate and two real insecticide substrates. The results suggest that carboxylesterase 001D was successfully expressed in the *E. coli* system. This is the first production of active carboxylesterase in this insect species with the *E. coli* system. Our work will be useful in the future to naturally produce active proteins for further three-dimensional structure studies of carboxylesterases in this species.

In this work, the results of pyrethroid hydrolysis analysis show that the purified proteins of 001D can metabolize the two real insecticide substrates, *β*-cypermethrin and the fenvalerate *in vitro*, and it thus indicates that the carboxylesterase 001D is involved in detoxification in the susceptible WH strain. In our earlier report, eight carboxylesterases, including the 001D from the susceptible GR strain, were shown to not increase the pyrethroid hydrolysis by mutation in the active sites (oxyanion hole and acyl-binding pocket) of the enzyme [29,30], suggesting that pyrethroid resistance in *H. armigera* may not occur through the first mechanism. Our results show the 001D can catalyze the pyrethroid substrates with a slow velocity, which probably suggests the overexpression of the carboxylesterase genes is indispensable to allow carboxylesterases to confer resistance to pyrethroid insecticides in *H. armigera*.

However, among the three fusion proteins, the fusion protein 6×His/S-tag/001D (pET30a) was expressed at a higher level compared to other two fusion proteins, Trx/6×His/S-tag/001D (pET32a) and 6×His/001D (pE1), and after a single step of Ni^2+^-NTA affinity purification, pET30a-001D showed a high purity as only a single band was observed in the SDS-PAGE and Western blot assay (Figure 3). Its hydrolytic activities towards pyrethroid insecticides are also higher compared to the other two fusion proteins (Table 2). The results suggest that S-tag has significantly improved expression of the esterase, but the larger fusion protein Trx-tag has little effect on improving expressionof the esterase in *E. coil*. In addition, the larger Trx-tag fused with the 001D may also hinder the carboxylesterase’s catalysis of the real insecticides, as the pyrethroid hydrolase activity of 001D from PET32a was lower than the 001D from PET30a. For each fusion protein, the variation between the esterase activity towards the model substrate (1-NA) and the hydrolytic activity towards the real insecticides (pyrethroids) are mainly due to the differences in their chemical structure between the model substrate and the real insecticides [15,16,43].

In most cases, enzyme activity can be easily influenced by the variation of temperature and pH value due to each enzyme only functioning efficiently within a suitable temperature and pH range. In the present study, the purified enzyme 001D showed high activity and stability over a broad range of temperatures. Its residual activity was 46.8% when it was incubated at 45 °C for 1h. It appears more stable than the carboxylesterase CpCE-1 from *Cydia pomonella* [36]. However, the esterase activity significantly decreased at a pH lower than 7.0. The effects of pH were similar to another esterase [44].

We also noted that the pyrethroid hydrolase activities of the carboxylesterase 001Dof the susceptible *H. armigera* WH strain expressed in *E. coli* are different from those of the carboxylesterase 001D of the susceptible GR strain from Australia and the susceptible YG strain from China, expressed in the baculovirus system [29,30,31]. By comparison, the hydrolase activity of 001D from the WH strain against *β*-cypermethrin is lower than that of the 001D from the GR strain, but it is to some extent closer to that of 001D from the YG strain (the specific activity is about 0.76 and 2.60 μM·min^−1^·(μM^−1^·protein)) towards the insecticidal pyrethroid isomers, 1*R trans*-α*S* and 1*R cis*-α*S*, respectively [31]. For the fenvalerate substrate, the specific activity of 001D from WH strain is 0.67 μM·min^−1^·(μM^−1^·protein),which is between the GR strain (0.81 μM·min^−1^·(μM^−1^·protein)) and the YG strain (0.0 μM·min^−1^·(μM^−1^·protein)) [31]. The variations of the pyrethroid hydrolase activity may be due to the recombinant esterase produced in different expression systems. In previous studies, the recombinant esterases were not tagged and expressed in insect cells by using a baculovirus system, and the assays were done with insect cell free extracts (cell lysis without purification) [29,31]. In the present study, the carboxylesterase was tagged and expressed in the *E. coli* cells, and the activity assays were performed using highly purified fusion proteins. We further observed that different fusion strategies have effects on protein expression and enzymatic activity. Furthermore, in previous work, the insecticides used in the hydrolytic activity assay were single isomers of cypermethrin and fenvalerate, but in this work both *β*-cypermethrin and fenvalerate substrates are in a complex of four isomers.

## 4. Materials and Methods

### 4.1. Chemicals and Plasmids

**Figure 5:**
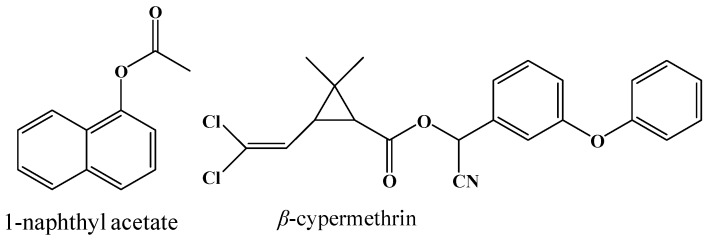

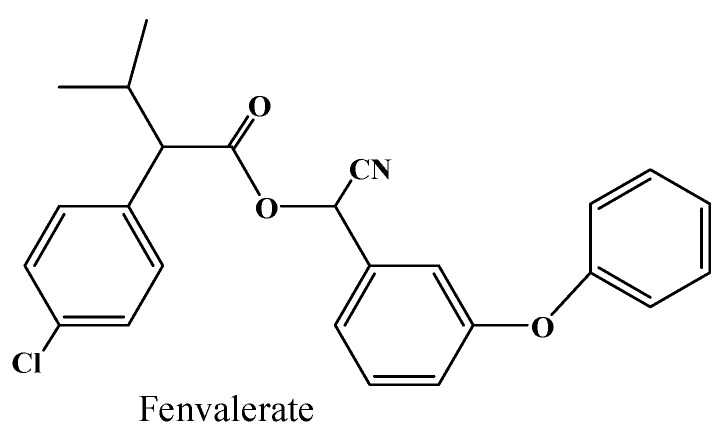
Structures of 1-naphthyl acetate, fenvalerate, and β-cypermethrin used in this paper.

The insecticides *β*-cypermethrin (comprising: 1*S trans*-α*R*, 1*S cis*-α*R*, 1*R trans*-α*S*, 1*R cis*-α*S*) and fenvalerate (Figure 5) were of analytical standard and provided by Aladdin Industrial Corporation (Shanghai, China). 1-naphthyl acetate and Fast Blue RR salt were also purchased from Aladdin Industrial Corporation. Protein molecular mass marker was purchased from Takara (Kusatsu, Japan) and Sangon (Shanghai, China). The pGEM-T easy vector was provided by Promega (Madison, WI, USA), *pEASY*^®^-Blunt E1 vector (pE1) was obtained from TransGen Biotech (Beijing, China), pET30a and PET32a were purchased from Novagen (Darmstadt, Germany ).

### 4.2. Insects

The susceptible *H. armigera* strain from the Wuhan region of China (WH strain) was originally collected from the Wuhan region of the Hubei Province in China over 30 years ago and was maintained on an artificial diet without outcrossing at 28 °C ± 1 °C, 60% ± 10% relative humidity, and a photoperiod of 16:8 (Light:Dark). Adult moths were held under the same temperature and supplied with a 5% sugar solution.

### 4.3. Sequencing of 001D from the H. armigera Wuhan (WH) Strain

Total RNA was extracted from the midguts of fifth-instarlarvae by using the SV Total RNA Isolation System Kit (Promega). First-strand cDNA was synthesized from 1 μg of DNA-free total RNA, using RevertAid M-MuL V Reverse Transcriptase (Thermo Scientific, Waltham, MA, USA). Primers (HdF1: 5′CTGTATTAACTGGACTAGC3′; HdR1: 5′CGTTGCGCAGATAACTC3′) for amplifying the open reading frame (ORF) of the gene were designed based on the sequence deposited in NCBI database (GenBank^®^ accession number FJ997295). The 50 μL PCR reaction mixture contained 2 μL of template cDNA, 3.0 μL of 10 μM of each primers, 4.0 μL of 2.5 mM dNTP, 0.5 μL of PrimeSTAR HS DNA Polymerase, and 10 μL of 5× PrimeSTAR Buffer (Takara). The thermocycler program was 98 °C for 30 s, followed by 30 cycles of 98 °C for 12 s, 60 °C for 25 s, 72 °C for 2 min, and with a final extension of 72 °C for 10 min. The PCR products were gel-purified, ligated into the pGEM-T easy vector after an adenine was added at the 3′ terminus, and used to transform JM109 cells. White clones were sequenced (Invitrogen™, Shanghai, China) and were found to contain a *001D*cDNA that differed by 11 amino acid residues from the closest homologue in the database. The sequence of this new allele was deposited in the NCBI database (GenBank^®^ accession number KT345935).

### 4.4. Sequence Analysis

The sequences of carboxylesterase from *H. armigera* were aligned using CLUSTALW2 software [45]. Signal peptides for secretion were detected with the Signal P 4.1 server [46]. The molecular weight and theoretical isoelectric point were predicted using the ExPASy web tool [47].

### 4.5. Cloning and Expression of Recombinant Proteins

**Figure 6:**
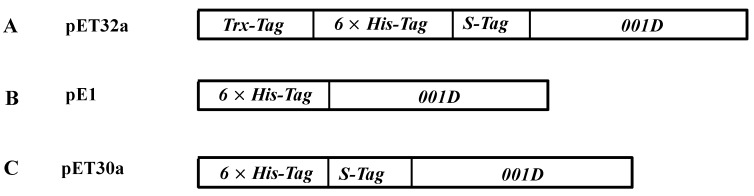
Gene maps of fusion recombinant plasmids harboring truncated (**A**) *Trx/6×His/S-tag/001D* from pET32a; (**B**) *6×His-tag/001D* from pE1; and (**C**) *6×His/S-tag/001D* from pET30a.

The carboxylesterase ORF encoding the mature enzyme lacking a signal peptide sequence was amplified with specific primer sequences (HdF2: 5′CCGGAATTCATGGACGACGAGTGGCGCGAGGTGAGGACT3′; HdR2: 5′GCGCTCGAGATTCTACAACTCGCTGCGTGGTCTGGGCGG3′) containing *EcoR*I and *Xho*I restriction endonuclease recognition sites (underlined). The amplicons were purified using the Universal DNA Purification Kit (Tiangen Biotech, Beijing, China), digested with the corresponding endonuclease and ligated into either pET30a or PET32a using T4 DNA ligase. Plasmids were verified by restriction digestion and DNA sequencing (Invitrogen™). The amplicons were also directly ligated into pE1 Expression Vector according to the manufacturer’s protocol, and then verified by sequencing. The plasmids (Figure 6) were transformed into the *E. coli* BL21 (DE3) to produce recombinant proteins, each containing a 6×His-tag at N-terminus for purification.

For protein expression, a colony from freshly transformed *E. coli* was used to inoculate 5 mL of Luria-Bertani (LB) broth medium containing 50 μg·mL^−1^ kanamycin. Cells were cultured overnight at 37 °C with shaking at 220 rpm. 4 mL cell suspension was used to inoculate 400 mL of LB medium containing 1% casein hydrolysate (Oxoid, Basingstoke, UK), 17 mM KH_2_PO_4_ and 72 mM K_2_HPO_4_, 50 μg·mL^−1^ kanamycin [48], and induced with 0.2 mM isopropyl β-d-thiogalactopyranoside (IPTG) for 48 h at 18 °C with shaking at 200 rpm. Cells were harvested when cell growth reached the exponential phase (OD_600_ = 0.5–0.6).

### 4.6. Purification of Expressed Carboxylesterases and Western Blot Analysis

Bacterial cells were harvested by centrifugation and stored at −70 °C until further processing. Cell pellets were re-suspended in 25 mM Tris-HCl (pH 8.0) at a concentration of 1 mL per 200 mg cell wet weight and then lysed by sonication. Supernatant was harvested after centrifugation at 17,400× *g* (Himac CR22G, Hitachi Ltd., Tokyo, Japan) at 4 °C for 30 min and further clarified by passing through a 0.22 μm filter. For purification of 6×His-tagged recombinant proteins, 1 mL of Ni^2+^-NTA agarose gel column (TransGen Biotech) was equilibrated with 10 mL of balance buffer (10 mM Tris-HCl, pH 8.0, 300 mM NaCl, 50 mM NaH_2_PO_4_, and 10 mM imidazole). Supernatant was added to the column and then washed with 6 mL of balance buffer. Bound proteins were eluted from the affinity resin with 5 mL elution buffer (balance buffer containing a linear gradient of 50–250 mM imidazole). Fractions were collected and analyzed on a 12% SDS-PAGE gel and stained with Coomassie Blue R250 solution (Aladdin Industrial Corporation). The recombinant fusion protein was then dialyzed against 25 mM Tris-HCl (pH 8.0), and aliquots (50–100 μL) were stored at −70 °C for later use. The purified target proteins were verified via Western blot analysis with anti-6×His monoclonal antibody (Protein Tech, Chicago, IL, USA), followed by staining with goat anti-mouse IgG horseradish peroxides (HRP) conjugate (Pierce Biotechnology, Rockford, IL, USA). Antibody binding was detected with a diaminobenzidine kit according to the manufacturer’s protocol (OriGene, Beijing, China). Protein concentrations were determined using the Bradford method with bovine serum albumin as a standard [49].

### 4.7. Assay of Enzymatic Activity

The kinetics of carboxylesterase activities against 1-naphthyl acetate were determined by the method of Han *et al.* [12] and Teese *et al.* [29] with a modification. Reactions were carried out in a 96-well microplate at 30 °C in a final volume of 200 μL in 0.1 M sodium phosphate buffer (pH 7.0), unless otherwise described. Each well contained 100 μL of appropriately diluted purified enzyme (0.6–1.75 μg) in buffer, 100 μL of 1-naphthyl acetate in buffer containing 2% (*v*/*v*) ethanol and 3 mM Fast Blue RR salt at a range of substrate concentrations (16–400 μM·L^−1^) spanning the *K*m (based on preliminary assays). Formation of 1-naphthol was monitored by recording the change in absorbance at 450 nm for 10 min in a microplate reader (M200 PRO, Tecan, Männedorf Switzerland) and quantified using 1-naphthol standard curves. Values for *k*cat and *K*m were estimated using “Hyper32” hyperbolic regression software [50].

Assays for the hydrolysis of *β*-cypermethrin and fenvalerate were carried out by monitoring substrate loss by a reversed-phase high performance liquid chromatography unit (HPLC 600 Controller, Waters Co., Milford, MA, USA) attached to a dual λ absorbance detector (Waters 2487) as described by Teese *et al.* [29] with some modification. The reaction mix contained 50 μL of 200 μM·L^−1^ β-cypermethrinorfenvalerate and 50 μL of diluted purified enzyme (1.75–2.4 μg). Reactions were stopped by the addition of 100 μL of acetonitrile and vortexing. Samples were then prepared by passing through a 0.22 μm filter before transferring into a 2 mL brown sample vial (Agilent, Santa Clara, CA, USA). An aliquot (10 μL) of each reaction was injected on a Symmetry C18 column (4.6 mm × 250 mm, 5 μm) and monitored at 210 nm, the mobile phase contained a mixture of acetonitrile and water (77:23, *v*/*v*) with a 1.0 mL·min^−1^ flow rate. For all substrates, control reactions were performed with the purified enzyme of 001D from pET30a inactivated by boiling for 10 min.

### 4.8. Thermostability and pH Stability

The optimal temperature of the enzyme was investigated by incubating the enzyme and substrate (1-naphthyl acetate) at different temperatures (15 °C–60 °C) in 100 mM phosphate buffer (pH 7.0) for 5 min. The thermostability of the enzymes was determined by incubating the purified recombinant protein at temperatures from 15 to 60 °C for 1 h. The residual activity was measured and expressed as a percentage of the highest activity obtained in this case. The optimal pH of the enzyme activity was determined using the following 25 mM buffers: citric acid buffer (pH 4.0–6.0), phosphate buffer (pH 7.0) and Tris-HCl buffer (pH 8.0–11.0). The determination of pH stability was performed by pre-incubating the enzyme in a corresponding buffer for 1 h at 30 °C. Then, enzyme activity was measured as above in 100 mM phosphate buffer (pH 7.0). The fusion protein 6×His/S-tag/001D (pET30a) was used in all above assays. The highest activity obtained in this casewas used as the control (100%).

## 5. Conclusions

Here we cloned the carboxylesterase *001D* gene from the susceptible *H. armigera* WH strain, and it contains the highly-conserved residues of catalytic triad and other active subsites in its amino acid sequence. We successfully expressed the carboxylesterase 001D fused with three different solubility/affinity tags in the *E. coli* cells. We purified the fusion proteins and evaluated their hydrolytic activities towards a model substrate and two pyrethroid insecticides. The results showed that carboxylesterase 001D was active and involved in detoxification, which probably implicated that overexpression of the genes to allow for effective sequestration of the insecticides is necessary to allow carboxylesterases to confer resistance to these insecticides in *H. armigera*.

## Figures and Tables

**Table 1:** Kinetic parameters for the purified carboxylesterases towards the 1-naphthyl acetate.

Enzyme	Specific Activity	*K*m	*k*cat
pET32a-001D	2.24(0.14)	7.61(0.63)	2.29(0.08)
pE1-001D	0.34(0.14)	19.72(6.69)	0.35(0.05)
pET30a-001D	0.57(0.07)	12.51(2.14)	0.60(0.06)

Specific activities (at 200 µM substrate) (μM·s^−1^·(μM^−1^·protein)) and estimates of *K*m (µM) and *k*cat (s^−1^) are shown. Estimates are based on an average of three replicates, and standard errors for these estimates are given in brackets.

**Table 2:** Specific activities of purified fusion carboxylesterases expressed in *E. coli* towards pyrethroid insecticides.

Enzyme	*β*-Cypermethrin	Fenvalerate
pET32a-001D	0.15(0.02)	0.32(0.02)
pE1-001D	0.19(0.02)	0.13(0.06)
pET30a-001D	0.41(0.04)	0.67(0.17)
Control	0.10(0.01)	0.03(0.02)

The values shown are micromoles of substrate hydrolyzed per micromole of enzyme per minute under the conditions of the assay. They are means with standard error sbased on an average of four replicates. For all substrates, control reactions were performed with the purified enzyme of pET30a-001D inactivated by boiling for 10 min.

## Abbreviations

1-NA	1-naphthyl acetate
OP	Organophosphate
CCE	Carboxy/cholinesterase(E.C.3.1.1)
PAGE	Polyacrylamide gel electrophoresis
pE1	*pEASY*^®^-Blunt E1 vector
